# Peritoneal metastasis of high-grade glioma via Ventriculoperitoneal shunt

**DOI:** 10.1093/omcr/omaf080

**Published:** 2025-06-27

**Authors:** Hawro T Hamza, Huda S Sulaiman, Muazzaz G Jaafar, Savan S Shengola, Bahkan J Saeed, Sami S Omar, Fahmi M Fatah, Kakil I Rasul, Saadi A Surchi, Fayrooz A Kakasur, Mohammed N Gheni, Chinar A Mustafa

**Affiliations:** Department of Oncology, Nanakali Teaching Hospital, Khabat Street, Azadi Quarter, Erbil 44001, Kurdistan Region, Iraq; Department of Oncology, Nanakali Teaching Hospital, Khabat Street, Azadi Quarter, Erbil 44001, Kurdistan Region, Iraq; Department of Radiology, Nanakali Teaching Hospital, Khabat Street, Azadi Qaurter, Erbil 44001, Kurdistan Region, Iraq; Department of Anatomy and Pathology, College of Medicine, Hawler Medical University, Erbil 44001, Kurdistan Region, Iraq; Department of Oncology, Nanakali Teaching Hospital, Khabat Street, Azadi Quarter, Erbil 44001, Kurdistan Region, Iraq; Rizgary Teaching Hospital, Rizgary Oncology Center, Koya Street, Rizgary District, Erbil 44001, Kurdistan Region, Iraq; Rizgary Teaching Hospital, Rizgary Oncology Center, Koya Street, Rizgary District, Erbil 44001, Kurdistan Region, Iraq; National Center for Cancer Care and Research (NCCCR), Hamad Medical Corporation (HMC), NCCCR, Doha 00000, Qatar; Weill Cornell Medicine- Qatar (WCM-Q), WCM-Q, Doha 00000, Qatar; Department of Neurosurgery, Hawler Teaching Hospital, Erbil 44001, Kurdistan Region, Iraq; Department of Histopathology, Rizgary Teaching Hospital, Koya Street, Rizgary District, Erbil 44001, Kurdistan Region, Iraq; Department of Digestive Surgery, Rizgary Teaching Hospital, Koya Street, Rizgary District, Erbil 44001, Kurdistan Region, Iraq; Department of Surgery, Rizgary Teaching Hospital, Koaya Street, Rizgary District, Erbil 44001, Kurdistan Region, Iraq

**Keywords:** glioma, Ventriculoperitoneal shunt, Extraneural metastasis, peritoneal metastasis, case report

## Abstract

High-grade gliomas (HGGs) are aggressive primary brain tumors with a poor prognosis. Although extracranial metastasis is uncommon, peritoneal dissemination via ventriculoperitoneal (VP) shunts is extremely rare. Here, we describe the case of an 18-year-old female with HGG who developed peritoneal metastasis one year after VP shunt placement. Although VP shunting in the presence of an intracranial high-grade tumor is generally not contraindicated, shunt-related metastasis should be recognized as a potential risk and an important, albeit rare, clinical presentation. This case highlights the diagnostic challenges and therapeutic limitations of this rare complication. Understanding this phenomenon is crucial for developing effective monitoring and treatment strategies.

## Introduction

High-grade gliomas (HGGs), the most common and aggressive primary brain tumors in adults, include anaplastic astrocytoma (grade 3) and glioblastoma (grade 4) according to the 2021 World Health Organization (WHO) classification, with survival typically ranging from 2–3 years for anaplastic astrocytoma and 8–15 months for GBM [1].

Primary central nervous system (CNS) tumors can spread beyond the brain, meninges, and spinal cord to form extraneural metastases (ENM); however, this is a very rare event, occurring in less than 2% of cases, although not fully understood, likely due to the short survival of affected patients and biological barriers that prevent tumor cells from thriving outside the CNS [2].

The most frequent sites of ENM are the lungs, pleura, lymph nodes, bones, liver, heart, and adrenal [3].

However, metastasis to the peritoneal cavity, particularly in the context of a VP shunt, is extremely uncommon [4].

HGGs are primarily evaluated with MRI with contrast, the gold standard for assessing tumor size, and location. Advanced techniques like MR perfusion and MR spectroscopy help differentiate tumor grades and assess metabolism. PET imaging aids in detecting recurrence and metabolic activity, while CT scans are used in patients who cannot have an MRI [5].

The VP shunt is a surgical intervention frequently employed to alleviate hydrocephalus in patients with HGG and inadvertently provides a potential route for tumor cells to escape the CNS and implant within the peritoneal cavity [6].

## Case report

**Figure 1:**
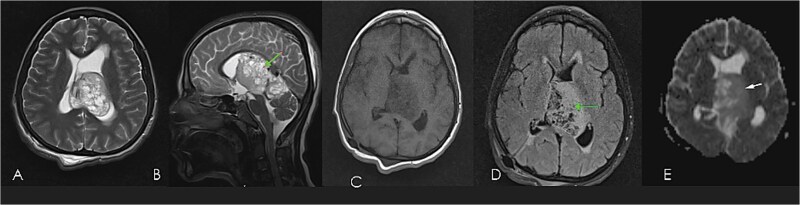
Pre-operative brain MRI of diffuse astrocytoma: T2WI axial (A), & sagittal (B), T1WI axial (C), FLAIR axial (D), ADC axial (E) images showing bubbly heterogeneously enhancing mass (enhanced images not shown), mixed signal intensity in T2WI, iso in T1WI, high on FLAIR, showing diffusion restriction on ADC; indicating highly cellular high-grade tumor involving deep white matter, extending to pineal and quadrigeminal cisterns causing mass effect with moderate hydrocephalous.

An 18-year-old female, with an unremarkable past history and family history of cancers, is presented with severe headache associated with nausea, vomiting, nystagmus, and diplopia for a 1-month duration. Brain MRI revealed a large heterogeneous mass arising from the left thalamus and exophytic growth in the lateral ventricles. It was associated with the dilatation of both lateral ventricles in keeping with the obstruction of outflow at the foramen of Munro (Fig. 1).

**Figure 2:**
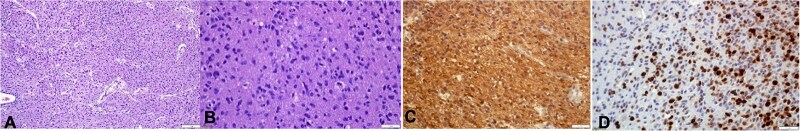
(A) Brain biopsy showed a diffuse cellular lesion with medium power magnification. (B) Brain biopsy showed a cellular lesion featuring marked nuclear atypia and pleomorphism and fibrillary cytoplasm, high power magnification. (C) Brain biopsy, and immunohistochemical staining for GFAP showed a strong and diffuse pattern of stain of the neoplastic cells. (D) Brain biopsy, ki67 immunohistochemical staining showed a high proliferative index.

**Figure 3:**
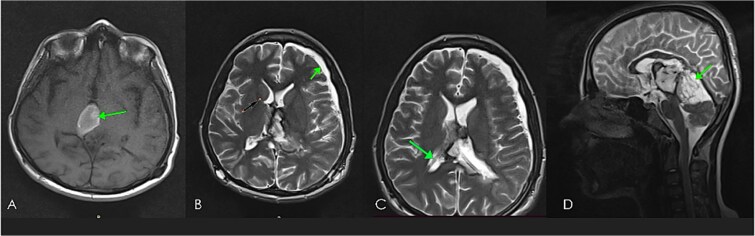
Early post-operative brain MRI images (A) axial T1WI without contrast, (B & C) axial T2WI; showing small hematoma at operative bed (green arrow in A), & small LT subdural hematoma (green arrow in B), with shunt tube seen at improper site at RT cauda-thalamic recess (black arrow in B), another functioning properly seated shunt tube seen within RT lateral ventricle sagittal T2WI in (D) showing residual cystic component of the mass (diffuse astrocytoma) green arrow in the Quadrigeminal and pineal region cisterns.

**Figure 4:**
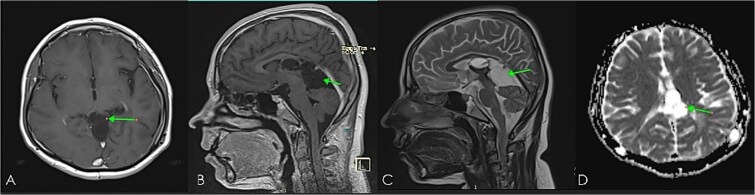
Follow-up brain MRI, axial (A), sagittal (B), post-contrast images, sagittal T2WI (C), and ADC map (D) showing non-enhancing cystic lesion with free diffusion representing residual disease and getting smaller in size in response to CCRT as compared with previous images. Note the resolution of early post-operative hematoma and subdural hemorrhage.

She underwent craniotomy with subtotal resection of the tumor, and immunohistochemistry (IHC) revealed a diffuse astrocytoma CNS WHO grade III, IDH-Wild (Fig. 2). Two weeks after the first operation, the patient developed obstructive encephalopathy and a VP shunt was inserted (Fig. 3). The shunt was obstructed and two other shunts were inserted (triple shunts). Postoperative complications and recovery from surgery lasted for three months. The patient received standard radiotherapy of 60 Gy in 30 fractions with concurrent and adjuvant temozolomide for 6 months. A follow-up MRI was performed 10 months postoperatively, suggesting stable disease, and she continued on regular surveillance (Fig. 4).

**Figure 5:**
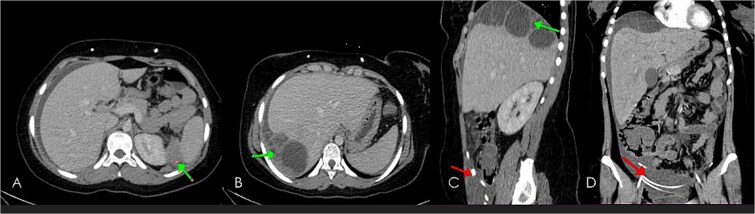
Computed tomography post contrast portal venous phase images axial (A, B), sagittal (C) and coronal reformat (D), showing large thick ascites as well as enhancing peritoneal cystic masses (green arrows) representing metastatic peritoneal lesions. Note the Ventriculoperitoneal shunts (in red arrows).

**Figure 6:**
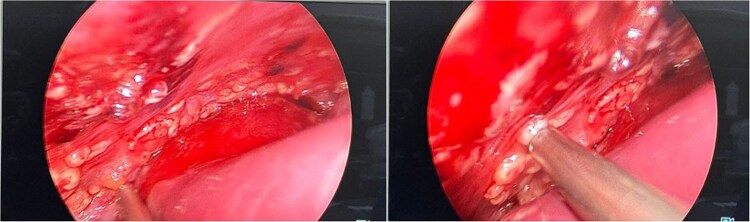
Diagnostic laparoscopy which showed multiple peritoneal and omental masses with huge ascites.

She developed severe bilateral loin pain, right hypochondrial pain, and low backache one year after diagnosis. A gynecological, urological, hepatopancreatobiliary, or shunt-related causes like malfunction, pseudocyst formation, or peritonitis were suspected, so she had abdominal ultrasonography which showed ascites and an intra-abdominal lesion, suspicious for a cyst or an abscess. CT scan of the chest, abdomen, and pelvis suggested peritoneal carcinomatosis (Fig. 5). The patient underwent diagnostic laparoscopy, which revealed multiple peritoneal and omental masses with huge ascites (Fig. 6), and biopsies were obtained.

**Figure 7:**
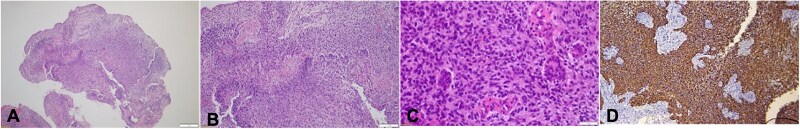
(A) A peritoneal biopsy showed a cellular spindle and low power magnification. (B) A peritoneal biopsy showed a cellular spindle lesion with areas of palisading necrosis and microvascular proliferation, medium power magnification. (C) Peritoneal biopsy showing cellular lesion with marked atypia and pleomorphism and foci of microvascular proliferation, high power magnification. (D) Peritoneal biopsy, and immunohistochemical staining for GFAP showed a strong and diffuse positivity of the neoplastic cells.

Peritoneal biopsy revealed a proliferating spindle cell lesion featuring marked nuclear atypia and pleomorphism, brisk mitotic activity, microvascular proliferation, and foci of palisading necrosis (Fig. 7). The tumor cells were positive for GFAP but negative for IDH1 on IHC; accordingly, a diagnosis of metastatic HGG was made.

**Figure 8:**
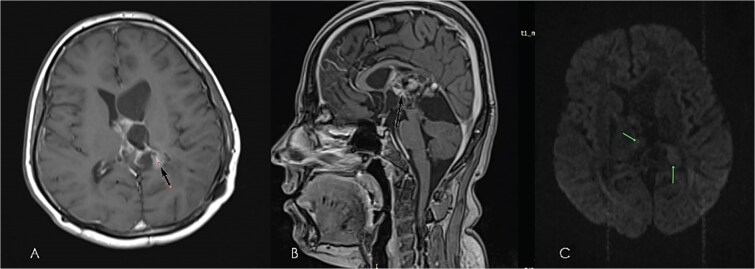
Further follow-up (14 months from operation) brain MRI: post-contrast images; axial (A), Sagittal (B), and diffusion-weighted image (C), showing disease progression with enlarging peripherally enhancing cystic lesion with mild diffusion restriction. Note: mass effect causing dilatation of frontal horn of LT lateral ventricle.

**Figure 9:**
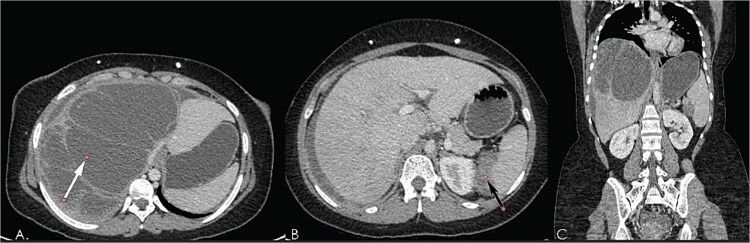
Computed tomography post-contrast portal venous phase images axial (A, B), and coronal reformat (C), showing interval progression in size of enhancing peritoneal cystic masses (white arrow in A, black in B) representing metastatic peritoneal lesions.

**Figure 10:**
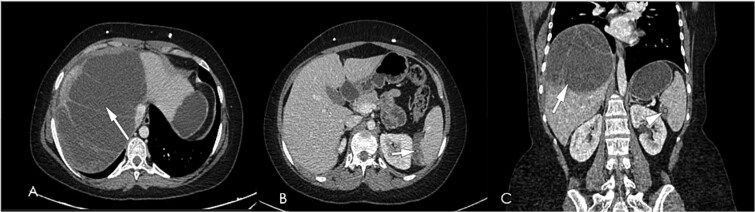
Computed tomography post-contrast portal venous phase images axial (A, B), and coronal reformat (C), dated august 2024; showing slight regression in size of enhancing peritoneal cystic metastatic lesions (white arrows in A & B, arrowhead in C) response less than 30%, regarded as stable disease per RECIST criteria.

She received 1^st^-line palliative chemotherapy (cisplatin and etoposide) for 2 months but she experienced disease progression per RECIST criteria in both the brain and intraperitoneal lesions (Figs. 8 and 9). Subsequently, she received 2^nd^-line treatment (bevacizumab plus irinotecan), her symptoms improved and she had stable disease for 9 months (Fig. 10).

## Discussion

This report presents a rare instance of peritoneal metastasis in HGG. While intracranial dissemination of glioma cells is well documented, extracranial spread, particularly to the peritoneum, is less common [3, 6].

To confirm ENM, Weiss suggested the following diagnostic criteria: (1) the metastatic lesion must be histologically consistent with a CNS tumor; (2) the clinical history must indicate a CNS tumor as the primary neoplasm; and (3) the primary and metastatic lesions must share identical morphological features [2].

Although the exact mechanism of ENM remains unclear, several theories have been proposed to explain the distant spread of intracranial tumors. It is believed to be facilitated by surgical interventions or vascular invasion, which compromise the blood–brain barrier, allowing hematogenous or lymphatic dissemination, as well as neoplastic cell shedding into the CSF and subsequent dissemination via shunts. Tumors near ventricles or basal cisterns are particularly prone to this behavior. Additionally, radiotherapy-induced sarcomatoid metaplasia and disruption of the blood–brain barrier may enhance the ability of tumor cells to invade the vessel walls [4, 6].

VP shunts are a significant risk factor for peritoneal metastases in high-grade gliomas [4, 7, 8]. Survival is poor, with reported outcomes ranging from 1 month [4] to 13 months [7].

The patient is initially treated according to the current practice guidelines [5].

Once the disease progressed, she had a good performance status however, in the absence of specific guidelines and no established consensus on optimal systemic therapy, treatment approaches are generally extrapolated from standard glioma management protocols [9]. However, the disease progressed within a short period with increasing ascites and abdominal pain. The patient was willing to receive additional treatment, after discussing the case again in the MDT, and bevacizumab, has been utilized in recurrent or progressive cases to potentially delay disease progression [5], so she received (bevacizumab plus irinotecan) regimen [10] and had stable disease for several months.

This report highlights the rare risk of peritoneal metastasis in primary brain HGG after VP shunt placement and the need for careful monitoring and a multidisciplinary approach. Preventative strategies like endoscopic third ventriculostomy instead of VP shunt should be explored, especially in patients with tumors near the ventricles [7]. Post-shunting surveillance, including routine abdominal imaging, is crucial. By detailing its clinical course, diagnostic challenges, and treatment, we aim to contribute to the literature and support improved management strategies. Further research is essential to enhance outcomes.

## References

[1] Wu J, Al-Zahrani A, Beylerli O. et al. Circulating miRNAs as diagnostic and prognostic biomarkers in high-grade gliomas. Front Oncol 2022;12:898537. 10.3389/fonc.2022.898537 35646622 PMC9133847

[2] Prabhakaran N, Miller DC, Litofsky NS. et al. Extraneural metastasis of primary glioma occurring in a setting of occupational ionizing radiation exposure. Case Rep Neurol Med 2019;2019:1748739. 31312534 10.1155/2019/1748739 PMC6595336

[3] Pasquier B, Pasquier D, N'golet A. et al. Extraneural metastases of astrocytomas and glioblastomas: Clinicopathological study of two cases and review of literature. Cancer 1980;45:112–25. 6985826 10.1002/1097-0142(19800101)45:1<112::aid-cncr2820450121>3.0.co;2-9

[4] Stephens S, Tollesson G, Robertson T. et al. Diffuse midline glioma metastasis to the peritoneal cavity via ventriculoperitoneal shunt: case report and review of literature. J Clin Neurosci 2019;67:288–93. 31266714 10.1016/j.jocn.2019.06.043

[5] National Comprehensive Cancer Network. Central Nervous System Cancers (Version 3.2024). Plymouth Meeting, PA: National Comprehensive Cancer Network; 2024. Available at: https://www.nccn.org/professionals/physician_gls/pdf/cns.pdf. Accessed October 7, 2024.

[6] Sun Q, Xu R, Xu H. et al. Extracranial metastases of high-grade glioma: the clinical characteristics and mechanism. World J Surg Oncol 2017;15:1–5. 28985756 10.1186/s12957-017-1249-6 PMC5639596

[7] Xu K, Khine KT, Ooi YC. et al. A systematic review of shunt-related extraneural metastases of primary central nervous system tumors. Clin Neurol Neurosurg 2018;174:239–43. 30292900 10.1016/j.clineuro.2018.09.038

[8] Newton HB, Rosenblum MK, Walker RW. Extraneural metastases of infratentorial glioblastoma multiforme to the peritoneal cavity. Cancer 1992;69:2149–53. 1311985 10.1002/1097-0142(19920415)69:8<2149::aid-cncr2820690822>3.0.co;2-g

[9] Massimino M, Spreafico F, Riva D. et al. A lower-dose, lower-toxicity cisplatin-etoposide regimen for childhood progressive low-grade glioma. J Neuro-Oncol 2010;100:65–71. 10.1007/s11060-010-0136-6 20151174

[10] Vredenburgh JJ, Desjardins A, Herndon JE. et al. Phase II trial of bevacizumab and irinotecan in recurrent malignant glioma. Clin Cancer Res 2007;13:1253–9. 17317837 10.1158/1078-0432.CCR-06-2309

